# A class of generalized invex functions and vector variational-like inequalities

**DOI:** 10.1186/s13660-017-1345-8

**Published:** 2017-04-07

**Authors:** Ru Li, Guolin Yu

**Affiliations:** Institute of Applied Mathematics, Beifang University of Nationalities, Yinchuan, Ningxia 750021 P.R. China

**Keywords:** 90C29, 90C46, 26B25, vector variational-like inequality, multi-objective programming, invex function, KKM-Fan theorem

## Abstract

In this paper, a class of generalized invex functions, called $(\alpha,\rho,\eta)$-invex functions, is introduced, and some examples are presented to illustrate their existence. Then we consider the relationships of solutions between two types of vector variational-like inequalities and multi-objective programming problem. Finally, the existence results for the discussed variational-like inequalities are proposed by using the KKM-Fan theorem.

## Introduction

As we all know, convexity and generalized convexity of functions play an important role in the field of optimization theory and its application. Among many generalized convexities given in the literature, a meaningful notion is called invex function, which was firstly introduced by Hanson [1]. Then Jeyakumar and Mond [2] popularized it and put forward the concept of *V*-invex function, and they studied the optimality conditions for nonlinear programming problem. Ivanov [3] provided the concept of second-order invex functions and dealt with the optimal solution of nonlinear programming problem. Yu and Liu [4] investigated a class of generalized invex type I functions and presented the optimality conditions of multi-objective programming. One aim of this paper is to present a new kind of generalized invex functions, termed $(\alpha,\rho,\eta)$-invex functions, which is weaker than the above mentioned generalized invexities.

It is well known that vector variational-like inequalities are of importance in the field of applied mathematics. There are several interesting and important topics, for instance, existence results of vector variational inequalities, which ensure the existence of efficient solutions of vector optimization problems and establish the relations between both problems. In order to do so, a great quantity of researchers have been attracted towards this direction, see [5–19]. In this note, we shall investigate the relations between vector variational-like inequalities and multi-objective programming problems under the hypothesis of $(\alpha,\rho,\eta)$-invexity. We also define a kind of generalized monotonicity functions, called $(\alpha,\rho,\eta)$-monotone functions. Then, by using the KKM-Fan theorem, we present the existence theorems for vector variational-like inequalities under the assumption of $(\alpha,\rho,\eta)$-monotonicity.

The content of the present work can be organized into four sections of which this introduction is the first. In Section 2, some notations and the concepts are recalled. Besides, a new class of generalized invex functions, called $(\alpha,\rho,\eta)$-invex functions, is introduced, and examples are provided in the support of this generalization. In Section 3, the relationships between Stampacchia and Minty invex vector variational-like inequalities and $(\alpha,\rho,\eta)$-invex multi-objective programming problem are discussed. Section 4 gives the existence theorems of solution of Stampacchia and Minty vector variational-like inequality under the hypothesis of $(\alpha,\rho,\eta)$-monotonicity.

## Notations and preliminaries

Throughout the paper, $\mathbb{R}^{n}$, $\mathbb{R}$, $\mathbb{R}_{+}$ and $\mathbb{R}_{++}$ represent the *n*-dimensional Euclidean space, the set of real numbers, the set of nonnegative real numbers and the set of positive real numbers, respectively. For any $x, y\in\mathbb{R}^{n}$, the inner product of *x* and *y* is denoted by $x^{T}y$. We use the following conventions for vectors in $\mathbb{R}^{n}$:

$$\begin{aligned} x= y \Leftrightarrow & x_{i}= y_{i},\quad \forall i= 1, \ldots,n; \\ x\leqq y \Leftrightarrow & x_{i}\leq y_{i},\quad \forall i=1,\ldots,n; \\ x\leqslant y \Leftrightarrow & x_{i}\leq y_{i},\quad \forall i=1,\ldots,n, i\neq j \mbox{ and }x_{j}< y_{i} \mbox{ for some } j; \\ x< y \Leftrightarrow & x_{i}< y_{i},\quad \forall i= 1, \ldots,n. \end{aligned}$$

We begin with recalling some known definitions, which will be applicable in the sequel of the paper. Suppose that $X\subseteq\mathbb{R}^{n}$ is a nonempty open subset and $\varphi:X\rightarrow\mathbb{R}$ is a real-valued function. In the rest of paper, we always assume that $\alpha,\rho: X\times X\rightarrow \mathbb{R}$ are real-valued functions and $\eta: X\times X\rightarrow \mathbb{R}^{n}$ is a vector-valued function.

### Definition 2.1

see [9]

The subset *X* is said to be invex with respect to $\eta: X\times X\rightarrow \mathbb{R}^{n}$ if for every $x, y\in X$, $\lambda\in[0, 1]$ it satisfies

$$ y+\lambda\eta(x, y)\in X. $$

In this case, *X* is called an *η*-invex set.

### Definition 2.2

see [9]

Let $\varphi: X\rightarrow \mathbb{R}$ be a function. The directional derivative of *φ* at $\bar{x}\in X$ in the direction of a vector $v\in X$, denoted by $\varphi'(\bar{x},v)$, is defined as

$$ \varphi'(\bar{x},v)=\lim_{t\rightarrow 0}\frac{\varphi(\bar{x}+tv)-\varphi(\bar{x})}{t}. $$

*φ* is called directionally differentiable at $\bar{x}\in X$ if the directional derivative of *φ* at *x̄* in any direction exists. *φ* is called directionally differentiable on *X* if it is directionally differentiable at every point of *X*.

### Definition 2.3

see [9]

Let $X\subseteq\mathbb{R}^{n}$ be an *η*-invex set and $\varphi: X\rightarrow \mathbb{R}$ be directionally differentiable on *X*. It is said that *φ* is *η*-invex at *x̄* if

$$ \varphi(x)-\varphi(\bar{x})\geq \varphi'\bigl(\bar{x},\eta(x, \bar{x})\bigr), \quad \forall x\in X. $$

*φ* is said to be *η*-invex on *X* if it is *η*-invex at every point of *X*.

Motivated by the above definition of *η*-invex function, we make two extensions of invexity to $(\alpha,\rho,\eta)$-invexity and pseudo-$(\alpha, \rho, \eta)$-invexity as follows.

### Definition 2.4

Let $X\subseteq\mathbb{R}^{n}$ be an *η*-invex set and $\varphi: X\rightarrow \mathbb{R}$ be directionally differentiable on *X*. It is said that *φ* is $(\alpha,\rho,\eta)$-invex at $\bar{x}\in X$ if there exist real-valued functions $\alpha, \rho: X\times X\rightarrow \mathbb{R}$ such that

2.1 $$ \varphi(x)-\varphi(\bar{x})\geq \alpha(x,\bar{x}) \varphi^{\prime}\bigl(\bar{x},\eta(x,\bar{x})\bigr)+\rho(x,\bar{x}),\quad \forall x\in X. $$

If *φ* is $(\alpha,\rho,\eta)$-invex for each $x\in X$, then *φ* is called $(\alpha,\rho,\eta)$-invex on *X*; *φ* is called strictly $(\alpha,\rho,\eta)$-invex at $\bar{x}\in X$ if equation (2.1) takes strict inequality, that is,

2.2 $$ \varphi(x)-\varphi(\bar{x})>\alpha(x,\bar{x})\varphi^{\prime} \bigl(\bar{x},\eta(x,\bar{x})\bigr)+\rho(x,\bar{x}),\quad \forall x\in X, x\neq \bar{x}. $$

If *φ* is strictly $(\alpha,\rho,\eta)$-invex for each $x\in X$, then *φ* is called strictly $(\alpha,\rho,\eta)$-invex on *X*.

### Remark 2.1

Setting $\alpha=1$ and $\rho=0$ in Definition 2.4, we arrive at the notion of *η*-invexity, defined by Farajzadeh and Lee (see [9]).

### Definition 2.5

Let $X\subseteq\mathbb{R}^{n}$ be an *η*-invex set and $\varphi: X\rightarrow \mathbb{R}$ be directionally differentiable on *X*. It is said that *φ* is $(\alpha, \rho, \eta)$-pseudoinvex at $\bar{x}\in X$ if there exist real-valued functions $\alpha, \rho: X\times X\rightarrow \mathbb{R}$ such that

$$ \alpha(x,\bar{x})\varphi^{\prime}\bigl(\bar{x},\eta(x,\bar{x})\bigr)+ \rho(x,\bar{x})\geq 0\Rightarrow \varphi(x)-\varphi(\bar{x})\geq 0,\quad \forall x\in X $$

or equivalently,

$$ \varphi(x)-\varphi(\bar{x})< 0 \Rightarrow \alpha(x,\bar{x}) \varphi^{\prime}\bigl(\bar{x},\eta(x,\bar{x})\bigr)+\rho(x,\bar{x})< 0,\quad \forall x\in X. $$

If function *φ* is $(\alpha, \rho, \eta)$-pseudoinvex at every $x\in X$, then *φ* is called $(\alpha, \rho, \eta)$-pseudoinvex on *X*.

The following two examples illustrate the existences of $(\alpha,\rho,\eta)$-invex functions and $(\alpha, \rho, \eta )$-pseudoinvex functions, respectively.

### Example 2.1

Let $X=\{x=(x_{1},x_{2})\in\mathbb{R}^{2}: x_{2}>x_{1}>0\}\cup\{(0,0)\}$. For arbitrary vectors $x, y\in X$, $x=(x_{1},x_{2})$ and $y=(y_{1},y_{2})$, consider the functions $\varphi: X\rightarrow \mathbb{R}$, $\alpha: X\times X\rightarrow \mathbb{R}$, $\rho: X\times X\rightarrow\mathbb{R}$, $\eta: X\times X\rightarrow \mathbb{R}^{n}$, defined by

$$ \varphi(x)=-x_{2},\qquad \alpha(x,y)= \vert x_{2}-y_{2} \vert +\frac{1}{10},\qquad \rho(x,y)=-(x_{1}+y_{2})^{2}, \qquad \eta(x,y)=(1,1). $$

By a direct calculation, we get that

$$ \varphi(y)-\varphi(x)=x_{2}-y_{2},\qquad \varphi^{\prime} \bigl(x,\eta(y,x)\bigr)=-1. $$

Therefore,

$$ \alpha(y,x)\varphi^{\prime}\bigl(x,\eta(y,x)\bigr)+\rho(y,x)=-\biggl( \vert y_{2}-x_{2} \vert +\frac{1}{10} \biggr)-(y_{1}+x_{2})^{2}. $$

If $\varphi(y)-\varphi(x)=x_{2}-y_{2}\geq 0$, then

$$ \alpha(y,x)\varphi^{\prime}\bigl(x,\eta(y,x)\bigr)+\rho(y,x)=- \biggl(x_{2}-y_{2}+\frac{1}{10}\biggr)-(y_{1}+x_{2})^{2} \leq 0. $$

Thus, it holds that

$$ \varphi(y)-\varphi(x)\geq \alpha(y,x)\varphi^{\prime}\bigl(x,\eta(y,x) \bigr)+\rho(y,x). $$

If $\varphi(y)-\varphi(x)=x_{2}-y_{2}< 0$, then

$$ \alpha(y,x)\varphi^{\prime}\bigl(x,\eta(y,x)\bigr)+\rho(y,x)=x_{2}-y_{2}- \frac{1}{10}-(y_{1}+x_{2})^{2}. $$

Thus, one has

$$\begin{aligned} \varphi(y)-\varphi(x)=x_{2}-y_{2} \geq & x_{2}-y_{2}-\frac{1}{10}-(y_{1}+x_{2})^{2} \\ = & \alpha(y,x)\varphi^{\prime}\bigl(x,\eta(y,x)\bigr)+\rho(y,x). \end{aligned}$$

So, we have verified that *φ* is an $(\alpha,\rho,\eta)$-invex function on *X*.

### Example 2.2

Let $X=\{x=(x_{1},x_{2})\in\mathbb{R}^{2}: x_{2}>x_{1}>0\}$. For arbitrary vectors $x, y\in X$, $x=(x_{1},x_{2})$ and $y=(y_{1},y_{2})$, consider the functions $\varphi: X\rightarrow \mathbb{R}$, $\alpha: X\times X\rightarrow \mathbb{R}$, $\rho: X\times X\rightarrow\mathbb{R}$, $\eta: X\times X\rightarrow \mathbb{R}^{n}$, defined by

$$ \varphi(x)=\frac{x_{2}}{x_{1}}, \qquad \alpha(x, y)=x_{1}y_{1}+x_{2}y_{2},\qquad \rho(x, y)=\frac{x_{1}y_{2}-x_{2}y_{1}}{x_{1}^{2}},\qquad \eta(x, y)=(x_{1},x_{2}). $$

By a direct calculation, we derive

$$\begin{aligned} &\varphi(y)-\varphi(x)=\frac{y_{2}}{y_{1}}-\frac{x_{2}}{x_{1}}=\frac{x_{1}y_{2}-x_{2}y_{1}}{x_{1}y_{1}}, \\ &\varphi^{\prime}\bigl(x, \eta(y, x)\bigr)=\lim_{t\rightarrow 0} \frac{\varphi(x+t\eta(y, x))-\varphi(x)}{t}=\frac{x_{1}y_{2}-x_{2}y_{1}}{x_{1}^{2}}. \end{aligned}$$

Therefore, it yields

$$\begin{aligned} \alpha(y, x)\varphi^{\prime}\bigl(x, \eta(y, x)\bigr)+\rho(y, x) =&(x_{1}y_{1}+x_{2}y_{2}) \frac{x_{1}y_{2}-x_{2}y_{1}}{x_{1}^{2}}+\frac{x_{1}y_{2}-x_{2}y_{1}}{x_{1}^{2}} \\ =& (x_{1}y_{1}+x_{2}y_{2}+1) \frac{x_{1}y_{2}-x_{2}y_{1}}{x_{1}^{2}}. \end{aligned}$$

If

$$ \varphi(y)-\varphi(x)=\frac{y_{2}}{y_{1}}-\frac{x_{2}}{x_{1}}=\frac{x_{1}y_{2}-x_{2}y_{1}}{x_{1}y_{1}}< 0, $$

then

$$ \alpha(y, x)\varphi^{\prime}\bigl(x, \eta(y, x)\bigr)+\rho(y, x)= (x_{1}y_{1}+x_{2}y_{2}+1) \frac{x_{1}y_{2}-x_{2}y_{1}}{x_{1}^{2}}< 0. $$

This shows that *φ* is $(\alpha, \rho, \eta )$-pseudoinvex on *X*.

In view of the concepts of monotonicities given by Al-Homidan *et al.* [20], we introduce $(\alpha, \rho, \eta )$-monotonicity for a real-valued function, which will be helpful in proving our results.

### Definition 2.6

Let $X\subseteq\mathbb{R}^{n}$, *α*, $\rho: X\times X\rightarrow \mathbb{R}$, $\eta:X\times X\rightarrow X$. The function $\varphi:X\rightarrow\mathbb{R}$ is said to be $(\alpha,\rho,\eta)$-monotone on *X* if

$$ \alpha(x,y)\varphi^{\prime}\bigl(y,\eta(x,y)\bigr)+\rho(x,y)+\alpha(y,x) \varphi^{\prime}\bigl(x,\eta(y,x)\bigr)+\rho(y,x)\leq 0 ,\quad \forall x,y\in X. $$

*From now on, unless otherwise specified, we always assume that* $X\subseteq\mathbb{R}^{n}$ *is an* *η-invex set,* $f(x)= (f_{1}(x),f_{2}(x),\ldots,f_{p}(x) )$ *,* $f_{i}:X \rightarrow \mathbb{R}$ *and* $i\in P=\{1,2,\ldots,p\}$ *.*

Consider the following *multi-objective programming problem* (MP):

$$ \mbox{(MP)}\qquad \textstyle\begin{cases} \mbox{minimize}& f(x)=(f_{1}(x),f_{2}(x),\ldots,f_{p}(x))\\ \mbox{subject to}& x\in X. \end{cases} $$

In multi-objective programming problems, objectives often conflict with each other. In this regard, the concepts of efficient and weak efficient solutions are widely used.

### Definition 2.7

A point $\bar{x}\in X$ is said to be an efficient solution of (MP) if there exists no $x\in X$ such that

$$ f_{i}(x)\leq f_{i}(\bar{x}),\quad \forall i \in P, $$

with strict inequality holding for at least one *i*.

### Definition 2.8

A point $\bar{x}\in X$ is said to be a weak efficient solution of (MP) if there exists no $x\in X$ such that

$$ f_{i}(x)< f_{i}(\bar{x}), \quad \forall i \in P. $$

Next, we are about to introduce the following Stampacchia and Minty vector variational-like inequalities, respectively, with also their weak formulations, which will be used to ensure the efficient solutions of the problem (MP). Let $\rho_{i}:X\times X \rightarrow \mathbb{R}$, $i\in P$.

(SVVI) For given real-valued functions *α* and $\rho_{i}$, $i\in P$, find $\bar{x}\in X$ such that there is no $x\in X$fulfilling

$$ \bigl(\alpha(x,\bar{x})f_{1}^{\prime}\bigl(\bar{x},\eta(x, \bar{x})\bigr) +\rho_{1}(x,\bar{x}),\ldots,\alpha(x, \bar{x})f_{p}^{\prime}\bigl(\bar{x},\eta(x,\bar{x})\bigr) + \rho_{p}(x,\bar{x})\bigr)\leqslant0. $$

(MVVI) For given real-valued functions *α* and $\rho_{i}$, $i\in P$, find $\bar{x}\in X$ such that there is no $x\in X$ fulfilling

$$ \bigl(\alpha(\bar{x},x)f_{1}^{\prime}\bigl(x,\eta(\bar{x},x) \bigr) +\rho_{1}(\bar{x},x),\ldots,\alpha(\bar{x},x)f_{p}^{\prime} \bigl(x,\eta(\bar{x},x)\bigr) +\rho_{p}(\bar{x},x)\bigr)\geqslant0. $$

(WSVVI) For given real-valued functions *α* and $\rho_{i}$, $i\in P$, find $\bar{x}\in X$ such that there is no $x\in X$ fulfilling

$$ \bigl(\alpha(x,\bar{x})f_{1}^{\prime}\bigl(\bar{x},\eta(x, \bar{x})\bigr) +\rho_{1}(x,\bar{x}),\ldots,\alpha(x, \bar{x})f_{p}^{\prime}\bigl(\bar{x},\eta(x,\bar{x})\bigr) + \rho_{p}(x,\bar{x})\bigr)< 0. $$

(WMVVI) For given real-valued functions *α* and $\rho_{i}$, $i\in P$, find $\bar{x}\in X$ such that there is no $x\in X$ fulfilling

$$ \bigl(\alpha(\bar{x},x)f_{1}^{\prime}\bigl(x,\eta(\bar{x},x) \bigr) +\rho_{1}(\bar{x},x),\ldots,\alpha(\bar{x},x)f_{p}^{\prime} \bigl(x,\eta(\bar{x},x)\bigr) +\rho_{p}(\bar{x},x)\bigr)>0. $$

### Remark 2.2

Let $\alpha \in \mathbb{R}_{++}$ and $\rho_{i}=0$ in above (SVVI) (respectively (WSVVI)), then this problem reduces to the vector variational inequality (respectively weak) introduced by Farajzadeh and Lee [9].

### Example 2.3

Let $X=\{x=(x_{1},x_{2})\in\mathbb{R}^{2}: x_{2}>x_{1}>0\}$ and $\bar{x}=(1,0)$. Consider the functions $f: X\rightarrow \mathbb{R}^{2}$, $\alpha: X\times X\rightarrow \mathbb{R}$ and $\rho_{i}: X\times X\rightarrow \mathbb{R}$, $i=\{1, 2\}$, defined by

$$f(x)=\bigl(f_{1}(x),f_{2}(x)\bigr), \qquad \alpha(x, \bar{x})=x_{1} \quad \mbox{and}\quad \bigl(\rho_{1}(x, \bar{x}), \rho_{2}(x, \bar{x})\bigr)=\biggl(\frac{2x_{2}}{x_{1}}, x_{1}x_{2}+1\biggr), $$

respectively, where

$$ f_{1}(x)=\frac{x_{2}}{x_{1}},\qquad f_{2}(x)=x_{2} ,\quad \forall x\in X. $$

Further, define $\eta(x, \bar{x})=x=(x_{1}, x_{2})$, $\forall x\in X$, then we have

$$\begin{aligned} f_{1}^{\prime}\bigl(\bar{x},\eta(x,\bar{x})\bigr) =&\lim _{t\rightarrow 0}\frac{f_{1}(\bar{x}+t\eta(x, \bar{x}))-f_{1}(\bar{x})}{t} \\ =&\lim_{t\rightarrow 0}\frac{f_{1}(1+tx_{1},tx_{2})-f_{1}(1,0)}{t} \\ =& \lim_{t\rightarrow 0}\frac{x_{2}}{1+tx_{1}} \\ =&x_{2} \end{aligned}$$

and

$$\begin{aligned} f_{2}^{\prime}\bigl(\bar{x},\eta(x,\bar{x})\bigr) =&\lim _{t\rightarrow 0}\frac{f_{2}(\bar{x}+t\eta(x, \bar{x}))-f_{2}(\bar{x})}{t} \\ =&\lim_{t\rightarrow 0}\frac{f_{2}(1+tx_{1},tx_{2})-f_{2}(1,0)}{t} \\ =& \lim_{t\rightarrow 0}\frac{tx_{2}}{t} \\ =&x_{2}. \end{aligned}$$

Therefore,

$$\begin{aligned} &\alpha(x,\bar{x})f_{1}^{\prime}\bigl(\bar{x},\eta(x,\bar{x}) \bigr) +\rho_{1}(x,\bar{x})=x_{1}x_{2}+ \frac{2x_{2}}{x_{1}}>0, \\ &\alpha(x,\bar{x})f_{2}^{\prime}\bigl(\bar{x},\eta(x,\bar{x}) \bigr) +\rho_{2}(x,\bar{x})=x_{1}x_{2}+x_{1}x_{2}+1=2x_{1}x_{2}+1>0. \end{aligned}$$

Thus, for every $x\in X$, we arrive at

$$ \bigl(\alpha(x,\bar{x})f_{1}^{\prime}\bigl(\bar{x},\eta(x, \bar{x})\bigr) +\rho_{1}(x,\bar{x}),\alpha(x,\bar{x})f_{2}^{\prime} \bigl(\bar{x},\eta(x,\bar{x})\bigr) +\rho_{2}(x,\bar{x})\bigr)>0, $$

which shows that *x̄* is a solution to (SVVI).

## The relationships between vector variational-like inequalities and multi-objective programming problems

In this section, we shall examine the relationships between Stampacchia and Minty vector variational-like inequalities and multi-objective programming problems in terms of $(\alpha,\rho,\eta)$-invex functions, which are formulated in Section 2.

### Theorem 3.1

*Let* *α*, $\rho_{i}: X\times X\rightarrow \mathbb{R}$, $\eta: X\times X\rightarrow \mathbb{R}^{n}$ *and for each* $i\in P$, $f_{i}$ *be* $(\alpha,\rho_{i},\eta)$-*invex function at* $\bar{x}\in X$. *If* *x̄* *solves* (SVVI), *then* *x̄* *is an efficient solution of* (MP).

### Proof

Suppose that *x̄* is not an efficient solution of (MP), then there exists $\hat{x}\in X$ such that

3.1 $$ f_{i}(\hat{x})-f_{i}(\bar{x})\leq0,\quad \forall i\in P, $$

and there exists at least one $i_{0}\in P$ such that

3.2 $$ f_{i_{0}}(\hat{x})-f_{i_{0}}(\bar{x})< 0. $$

Since $f_{i}$ is an $(\alpha,\rho_{i},\eta)$-invex function at $\bar{x}\in X$, it follows from Definition 2.4 that

3.3 $$ f_{i}(\hat{x})-f_{i}(\bar{x})\geq \alpha(\hat{x}, \bar{x})f_{i}^{\prime}\bigl(\bar{x},\eta(\hat{x},\bar{x})\bigr) + \rho_{i}(\hat{x},\bar{x}), \quad i\in P. $$

Combining inequalities (3.1), (3.2) and (3.3), we obtain that there exists $\hat{x}\in X$ such that

$$ \alpha(\hat{x},\bar{x})f_{i}^{\prime}\bigl(\bar{x},\eta(\hat{x}, \bar{x})\bigr) +\rho_{i}(\hat{x},\bar{x})\leq 0,\quad \forall i \in P, $$

and there is at least one $i_{0}\in P$ such that

$$ \alpha(\hat{x},\bar{x})f_{i_{0}}^{\prime}\bigl(\bar{x},\eta(\hat{x}, \bar{x})\bigr) +\rho_{i_{0}}(\hat{x},\bar{x})< 0. $$

This indicates that there exists $\hat{x}\in X$ such that

$$ \bigl(\alpha(\hat{x},\bar{x})f_{1}^{\prime}\bigl(\bar{x},\eta( \hat{x},\bar{x})\bigr) +\rho_{1}(\hat{x},\bar{x}),\ldots,\alpha( \hat{x},\bar{x})f_{p}^{\prime}\bigl(\bar{x},\eta(\hat{x},\bar{x}) \bigr) +\rho_{p}(\hat{x},\bar{x})\bigr)\leqslant 0, $$

which is a contradiction to the fact that *x̄* is a solution to (SVVI). □

### Theorem 3.2

*Let* *α*, $\rho_{i}: X\times X\rightarrow \mathbb{R}$, $\eta: X\times X\rightarrow \mathbb{R}^{n}$ *and for each* $i\in P$, $f_{i}$ *be an* $(\alpha,\rho_{i},\eta)$-*pseudoinvex function at* $\bar{x}\in X$. *If* *x̄* *solves* (WSVVI), *then* *x̄* *is a weak efficient solution of* (MP).

### Proof

We proceed by contradiction. Assume that *x̄* is not a weak efficient solution of (MP), then there exists $\hat{x}\in X$ such that

$$ f_{i}(\hat{x})-f_{i}(\bar{x})< 0,\quad \forall i\in P. $$

Because $f_{i}$ is $(\alpha,\rho_{i},\eta)$-pseudoinvex at $\bar{x}\in X$, we derive

$$ \alpha(\hat{x},\bar{x})f_{i}^{\prime}\bigl(\bar{x},\eta(\hat{x}, \bar{x})\bigr)+\rho_{i}(\hat{x},\bar{x})< 0,\quad \forall i\in P. $$

That is,

$$ \bigl(\alpha(\hat{x},\bar{x})f_{1}^{\prime}\bigl(\bar{x},\eta( \hat{x},\bar{x})\bigr) +\rho_{1}(\hat{x},\bar{x}),\ldots,\alpha( \hat{x},\bar{x})f_{p}^{\prime}\bigl(\bar{x},\eta(\hat{x},\bar{x}) \bigr) +\rho_{p}(\hat{x},\bar{x})\bigr)< 0, $$

which contradicts the hypothesis that *x̄* solves (WSVVI). □

### Theorem 3.3

*Let* *α*, $\rho_{i}: X\times X\rightarrow \mathbb{R}$, $\eta: X\times X\rightarrow \mathbb{R}^{n}$ *and for each* $i\in P$, $f_{i}$ *be an* $(\alpha,\rho_{i},\eta)$-*invex function on* *X*. *If* $\bar{x}\in X$ *is an efficient solution of* (MP), *then* *x̄* *solves* (MVVI).

### Proof

Suppose that *x̄* does not solve (MVVI), then there exists $\hat{x}\in X$ satisfying

$$ \bigl(\alpha(\bar{x},\hat{x})f_{1}^{\prime}\bigl(\hat{x},\eta( \bar{x},\hat{x})\bigr) +\rho_{1}(\bar{x},\hat{x}),\ldots,\alpha( \bar{x},\hat{x})f_{p}^{\prime}\bigl(\hat{x},\eta(\bar{x},\hat{x}) \bigr) +\rho_{p}(\bar{x},\hat{x})\bigr)\geqslant0, $$

that is,

3.4 $$ \alpha(\bar{x},\hat{x})f_{i}^{\prime}\bigl(\hat{x},\eta(\bar{x}, \hat{x})\bigr) +\rho_{i}(\bar{x},\hat{x})\geq 0,\quad \forall i \in P, $$

and there is at least one $i_{0}\in P$ such that

3.5 $$ \alpha(\bar{x},\hat{x})f_{i_{0}}^{\prime}\bigl(\hat{x},\eta(\bar{x}, \hat{x})\bigr) +\rho_{i_{0}}(\bar{x},\hat{x})>0. $$

Noticing that $f_{i}$ is $(\alpha,\rho_{i},\eta)$-invex on *X*, we have

3.6 $$ f_{i}(\bar{x})-f_{i}(\hat{x})\geq\alpha(\bar{x},\hat{x}) f_{i}^{\prime}\bigl(\hat{x},\eta(\bar{x},\hat{x})\bigr)+ \rho_{i}(\bar{x},\hat{x}),\quad \forall i \in P. $$

Together with inequalities (3.4), (3.5) and (3.6), it follows that there exists $\hat{x}\in X$ satisfying

$$ f(\hat{x})\leqslant f(\bar{x}), $$

which leads to a contradiction, that *x̄* is an efficient solution of (MP). □

### Theorem 3.4

*Let* *α*, $\rho_{i}: X\times X\rightarrow \mathbb{R}$, $\eta: X\times X\rightarrow \mathbb{R}^{n}$ *and for each* $i\in P$, $f_{i}$ *be an* $(\alpha,\rho_{i},\eta)$-*invex function on* *X*. *If* $\bar{x}\in X$ *solves* (WSVVI), *then* *x̄* *solves* (WMVVI).

### Proof

Suppose that *x̄* solves (WSVVI), then there exists no $x\in X$ such that

$$ \bigl(\alpha(x,\bar{x})f_{1}^{\prime}\bigl(\bar{x},\eta(x, \bar{x})\bigr) +\rho_{1}(x,\bar{x}),\ldots,\alpha(x, \bar{x})f_{p}^{\prime}\bigl(\bar{x},\eta(x,\bar{x})\bigr) + \rho_{p}(x,\bar{x})\bigr)< 0, $$

i.e., there exists no $x\in X$ such that

3.7 $$ \alpha(x,\bar{x})f_{i}^{\prime}\bigl(\bar{x},\eta(x,\bar{x}) \bigr)+\rho_{i}(x,\bar{x})< 0,\quad \forall i\in P. $$

Since $f_{i}$ is $(\alpha,\rho_{i},\eta)$-invex on *X*, one has

3.8 $$ f_{i}(x)-f_{i}(\bar{x})\geq \alpha(x,\bar{x})f_{i}^{\prime} \bigl(\bar{x},\eta(x,\bar{x})\bigr)+\rho_{i}(x,\bar{x}),\quad \forall x\in X , i\in P. $$

Similarly, we can derive

3.9 $$ f_{i}(\bar{x})-f_{i}(x)\geq\alpha(\bar{x},x)f_{i}^{\prime} \bigl(x,\eta(\bar{x},x)\bigr)+\rho_{i}(\bar{x},x),\quad \forall x\in X, i \in P. $$

Adding inequalities (3.8) and (3.9), we get

3.10 $$ \alpha(\bar{x},x)f_{i}^{\prime}\bigl(x,\eta(\bar{x},x)\bigr)+ \rho_{i}(\bar{x},x)\leq -\bigl[\alpha(x,\bar{x})f_{i}^{\prime} \bigl(\bar{x},\eta(x,\bar{x})\bigr)+\rho_{i}(x,\bar{x})\bigr]. $$

Combining inequalities (3.7) and (3.10), we derive that there exists no $x\in X$ such that

$$ \alpha(\bar{x},x)f_{i}^{\prime}\bigl(x,\eta(\bar{x},x)\bigr)+ \rho_{i}(\bar{x},x)>0,\quad \forall i\in P, $$

i.e., there exists no $x\in X$ satisfying

$$ \bigl(\alpha(\bar{x},x)f_{1}^{\prime}\bigl(x,\eta(\bar{x},x) \bigr)+\rho_{1}(\bar{x},x),\ldots,\alpha(\bar{x},x)f_{p}^{\prime} \bigl(x,\eta(\bar{x},x)\bigr)+\rho_{p}(\bar{x},x)\bigr)>0,\quad \forall i \in P, $$

which implies that *x̄* solves (WMVVI). □

### Theorem 3.5

*Let* *α*, $\rho_{i}: X\times X\rightarrow \mathbb{R}$, $\eta: X\times X\rightarrow \mathbb{R}^{n}$ *and for each* $i\in P$, $f_{i}$ *be a strictly* $(\alpha,\rho_{i},\eta)$-*invex function on* *X*. *If* $\bar{x}\in X$ *is a weak efficient solution of* (MP), *then* *x̄* *solves* (MVVI).

### Proof

Suppose that *x̄* is a weak efficient solution of (MP) but does not solve (MVVI), then there exists $\hat{x}\in X$ $(\hat{x}\neq\bar{x})$ satisfying

$$ \bigl(\alpha(\bar{x},\hat{x})f_{1}^{\prime}\bigl(\hat{x},\eta( \bar{x},\hat{x})\bigr) +\rho_{1}(\bar{x},\hat{x}),\ldots,\alpha( \bar{x},\hat{x})f_{p}^{\prime}\bigl(\hat{x},\eta(\bar{x},\hat{x}) \bigr) +\rho_{p}(\bar{x},\hat{x})\bigr)\geqslant 0, $$

or equivalently,

3.11 $$ \alpha(\bar{x},\hat{x})f_{i}^{\prime}\bigl(\hat{x},\eta(\bar{x}, \hat{x})\bigr) +\rho_{i}(\bar{x},\hat{x})\geq 0,\quad \forall i\in P, $$

and there is at least one $i_{0}\in P$ such that

3.12 $$ \alpha(\bar{x},\hat{x})f_{i_{0}}^{\prime}\bigl(\hat{x},\eta(\bar{x}, \hat{x})\bigr) +\rho_{i_{0}}(\bar{x},\hat{x})> 0. $$

Noticing that $f_{i}$ is strictly $(\alpha,\rho_{i},\eta)$-invex on *X*, we obtain

3.13 $$ f_{i}(\bar{x})-f_{i}(\hat{x})>\alpha(\bar{x}, \hat{x})f_{i}^{\prime}\bigl(\hat{x},\eta(\bar{x},\hat{x})\bigr)+ \rho_{i}(\bar{x},\hat{x}),\quad \forall i\in P. $$

By inequalities (3.11), (3.12) and (3.13), we get that there exists $\hat{x}\in X$ satisfying

$$ f_{i}(\bar{x})-f_{i}(\hat{x})> 0,\quad \forall i\in P, $$

which contradicts the fact that *x̄* is an efficient solution of (MP). □

### Theorem 3.6

*Let* *α*, $\rho_{i}: X\times X\rightarrow \mathbb{R}$, $\eta: X\times X\rightarrow \mathbb{R}^{n}$ *and for each* $i\in P$, $f_{i}$ *be an* $(\alpha,\rho_{i},\eta)$-*invex function on* *X*. *If* $\bar{x}\in X$ *is a weak efficient solution of* (MP), *then* *x̄* *solves* (WMVVI).

### Proof

Assuming that *x̄* does not solve (WMVVI), therefore, there exists $\hat{x}\in X$ such that

$$ \bigl(\alpha(\bar{x},\hat{x})f_{1}^{\prime}\bigl(\hat{x},\eta( \bar{x},\hat{x})\bigr) +\rho_{1}(\bar{x},\hat{x}),\ldots,\alpha( \bar{x},\hat{x})f_{p}^{\prime}\bigl(\hat{x},\eta(\bar{x},\hat{x}) \bigr) +\rho_{p}(\bar{x},\hat{x})\bigr)>0, $$

that is,

3.14 $$ \alpha(\bar{x},\hat{x})f_{i}^{\prime}\bigl(\hat{x},\eta(\bar{x}, \hat{x})\bigr) +\rho_{i}(\bar{x},\hat{x})> 0,\quad \forall i\in P. $$

Noticing that each $f_{i}$ is $(\alpha,\rho_{i},\eta)$-invex on *X*, we derive

3.15 $$ f_{i}(\bar{x})-f_{i}(\hat{x})\geq \alpha(\bar{x}, \hat{x})f_{i}^{\prime}\bigl(\hat{x},\eta(\bar{x},\hat{x})\bigr)+ \rho_{i}(\bar{x},\hat{x}),\quad \forall i\in P. $$

We conclude from (3.14) and (3.15) that there exists $\hat{x}\in X$ satisfying

$$ f_{i}(\bar{x})-f_{i}(\hat{x})> 0,\quad \forall i\in P, $$

which leads to a contradiction that *x̄* is the weak efficient solution of (MP). □

## The solution existence of vector variational-like inequalities

This section is devoted to a discussion of the existence of solutions for Stampacchia and Minty vector variational-like inequalities under the assumption of $(\alpha,\rho,\eta)$-monotonicity. Now, we assume that *Y* is a topological vector space, $X\subseteq\mathbb{R}^{n}$ is a convex set, and $f_{i}:X \rightarrow \mathbb{R}$, $i\in P$.

### Definition 4.1

see [21]

Let *E* be a nonempty subset of a topological vector space *Y*. A multifunction $\psi: E\rightarrow2^{Y}$ is a KKM mapping if for any finite subset $\{x_{1}, x_{2}, \ldots , x_{n}\}$ of *E*, it satisfies

$$ co\{x_{1}, x_{2}, \ldots , x_{n}\}\subset \bigcup _{j=1}^{n}\psi(x_{j}), $$

where $co\{x_{1}, x_{2}, \ldots , x_{n}\}$ denotes the convex hull of $\{x_{1}, x_{2}, \ldots , x_{n}\}$.

### Lemma 4.1

see [21] (KKM-Fan theorem)

*Let* *E* *be a nonempty convex subset of a topological vector space* *Y*, *and let* $\psi: E\mapsto 2^{Y}$ *be a KKM mapping with closed values*. *If there is a point* $x_{0}\in E$ *such that* $\psi(x_{0})$ *is compact*, *then*

4.1 $$ {\bigcap}_{x\in E}{\psi(x)}\neq \phi. $$

### Theorem 4.1

*Let* *α*, $\rho_{i}: X\times X\rightarrow \mathbb{R}$, $i\in P$, $\eta: X\times X\rightarrow \mathbb{R}^{n}$. *Assume that*

(i) *for each* $i\in P$, $-f_{i}$ *is* $(\alpha,\rho_{i},\eta)$-*monotone on* *X*;
(ii) $\alpha(x,y) \in \mathbb{R}_{++}$, $x\neq y$, $x,y\in X$. $\rho_{i}(x,y)\in \mathbb{R}_{+}$, $x,y\in X$;
(iii) *α* *and* $\rho_{i}$ *are affine functions with respect to their second argument such that* $\alpha(x,x)=0$ *and* $\rho_{i}(x,x)=0$, $\forall x\in X$;
(iv) *the set*-*valued map* $\Gamma: X\rightarrow2^{X}$ *defined by*
  $$\begin{aligned} \Gamma(x) =&\bigl\{ y\in X:\bigl(\alpha(x,y)f_{1}^{\prime}\bigl(y,\eta(x,y) \bigr)+\rho_{1}(x,y),\ldots, \\ & \alpha(x,y)f_{p}^{\prime} \bigl(y,\eta(x,y)\bigr)+\rho_{p}(x,y)\bigr)\nleqslant 0, \forall x\in X\bigr\} , \end{aligned}$$
  *is closed valued*;
(v) *there exist a nonempty compact set* $M\subset X$ *and a nonempty compact convex set* $N\subset X$ *such that for each* $y\in X\backslash M$, *there exists* $x\in N$ *such that* $y \notin \Gamma(x)$.

*Then* (SVVI) *is solvable on* *X*.

### Proof

Define a set-valued map $\hat{\Gamma}(x): X\rightarrow2^{X}$ as

$$\begin{aligned} \hat{\Gamma}(x) =&\bigl\{ y\in X: \bigl(\alpha(y, x)f_{1}^{\prime}\bigl(x,\eta(y, x)\bigr)+\rho_{1}(y, x), \ldots, \\ & \alpha(y, x)f_{p}^{\prime}\bigl(x, \eta(y, x)\bigr)+\rho_{p}(y, x)\bigr)\nleqslant 0, \forall x\in X\bigr\} . \end{aligned}$$

It is obvious that $x\in \Gamma(x)\cap\hat{\Gamma}(x)$. Therefore, $\Gamma(x)$ and $\hat{\Gamma}(x)$ are nonempty. Firstly, we will demonstrate that Γ̂ is a KKM map on *X*. Indeed, suppose to the contrary that $\hat{\Gamma}(x)$ is not a KKM map, then there exists $\{x_{1}, x_{2}, \ldots, x_{n}\}\subset X$, $t_{j}\geq0$, $j=1, 2, \ldots, n$, with $\sum_{j=1}^{n}t_{j}=1$ such that

$$ \bar{x}=\sum _{j=1}^{n}t_{j}x_{j} \notin\bigcup_{j=1}^{n}\hat{ \Gamma}(x_{j}). $$

Hence, for any $j=1, 2, \ldots, n$,

$$ \bigl(\alpha(\bar{x},x_{j})f_{1}^{\prime} \bigl(x_{j},\eta(\bar{x},x_{j})\bigr)+\rho_{1}( \bar{x},x_{j}),\ldots,\alpha(\bar{x},x_{j})f_{p}^{\prime} \bigl(x_{j},\eta(\bar{x},x_{j})\bigr)+\rho_{p}( \bar{x},x_{j})\bigr)\geqslant 0, $$

that is,

$$ \alpha(\bar{x},x_{j})f_{i}^{\prime} \bigl(x_{j},\eta(\bar{x},x_{j})\bigr)+\rho_{i}( \bar{x},x_{j})\geq0, \quad \forall i\in P, $$

and there is at least one $i_{0}\in P$ such that

$$ \alpha(\bar{x},x_{j})f_{i_{0}}^{\prime} \bigl(x_{j},\eta(\bar{x},x_{j})\bigr)+\rho_{i_{0}}( \bar{x},x_{j})>0. $$

Therefore, for all $i\in P$, $j=1,2,\ldots,n$, we have

$$\begin{aligned} 0 =&\alpha(\bar{x},\bar{x})f_{i}^{\prime}\bigl(x_{j}, \eta(\bar{x},x_{j})\bigr)+\rho_{i}(\bar{x},\bar{x}) \\ =&\alpha\Biggl(\bar{x},\sum_{j=1}^{n}t_{j}x_{j} \Biggr)f_{i}^{\prime}\bigl(x_{j},\eta( \bar{x},x_{j})\bigr)+\rho_{i}\Biggl(\bar{x},\sum _{j=1}^{n}t_{j}x_{j}\Biggr) \\ =& \sum_{j=1}^{n}t_{j}\bigl( \alpha(\bar{x},x_{j})f_{i}^{\prime} \bigl(x_{j},\eta(\bar{x},x_{j})\bigr)+\rho_{i}( \bar{x},x_{j})\bigr) \\ >&0. \end{aligned}$$

This is a contradiction. Therefore, Γ̂ is a KKM map on *X*. Afterwards, it is necessary to verify that $\hat{\Gamma}(x)\subset\Gamma(x)$, $\forall x\in X$. If $\bar{x}\notin \Gamma(x)$, then there exists $x\in X$ such that

4.2 $$ \alpha(x,\bar{x})f_{i}^{\prime}\bigl(\bar{x},\eta(x,\bar{x}) \bigr)+\rho_{i}(x,\bar{x}))\leq 0,\quad \forall i\in P, $$

with strict inequality holding for at least one *i*. Because $-f_{i}$ is $(\alpha,\rho_{i},\eta)$-monotone on *X*, for all $i\in P$ and $x \in X$, we have

$$ \alpha(x, \bar{x}) (-f_{i})^{\prime}\bigl(\bar{x}, \eta(x, \bar{x})\bigr)+\rho_{i}(x, \bar{x})+\alpha(\bar{x}, x) (-f_{i})^{\prime}\bigl(x, \eta(\bar{x}, x)\bigr)+ \rho_{i}(\bar{x}, x)\leq 0. $$

This leads to

$$ -\alpha(x, \bar{x})f_{i}^{\prime}\bigl(\bar{x}, \eta(x, \bar{x}) \bigr)+\rho_{i}(x, \bar{x})-\alpha(\bar{x}, x)f_{i}^{\prime} \bigl(x, \eta(\bar{x}, x)\bigr)+\rho_{i}(\bar{x}, x)\leq 0. $$

Thus,

$$ \alpha(x, \bar{x})f_{i}^{\prime}\bigl(\bar{x}, \eta(x, \bar{x}) \bigr)-\rho_{i}(x, \bar{x})+\alpha(\bar{x}, x)f_{i}^{\prime} \bigl(x, \eta(\bar{x}, x)\bigr)-\rho_{i}(\bar{x}, x)\geq 0. $$

Therefore, we arrive at

$$\begin{aligned} &\alpha(x, \bar{x})f_{i}^{\prime}\bigl(\bar{x}, \eta(x, \bar{x}) \bigr)+\rho_{i}(x, \bar{x})\\ &\quad \geq-\alpha(\bar{x}, x)f_{i}^{\prime} \bigl(x, \eta(\bar{x}, x)\bigr)+\rho_{i}(\bar{x}, x)+2 \rho_{i}(x, \bar{x}). \end{aligned}$$

Combining the above inequality with (4.2) yields

$$\begin{aligned} 0 \leq &\alpha(\bar{x}, x)f_{i}^{\prime}\bigl(x, \eta(\bar{x}, x)\bigr)-\rho_{i}(\bar{x}, x)-2\rho_{i}(x, \bar{x}) \\ \leq & \alpha(\bar{x}, x)f_{i}^{\prime}\bigl(x, \eta(\bar{x}, x)\bigr)+\rho_{i}(\bar{x}, x), \end{aligned}$$

with strict inequality holding for at least one *i*. So, we get that there exists $x\in X$ satisfying

$$ \bigl(\alpha(\bar{x}, x)f_{1}^{\prime}\bigl(x, \eta(\bar{x}, x) \bigr)+\rho_{1}(\bar{x}, x), \ldots,\alpha(\bar{x}, x)f_{p}^{\prime}\bigl(x, \eta(\bar{x}, x)\bigr)+ \rho_{p}(\bar{x}, x)\bigr)\geqslant 0. $$

Therefore, $\bar{x}\notin \hat{\Gamma}(x)$. Up to now, we have shown that $\hat{\Gamma}(x)\subset\Gamma(x) $ for any $x\in X$. Hence, Γ is also a KKM map. From hypotheses (iv) and (v), we know that $\Gamma(x)$ is a closed subset of the compact set. Thus, we derive that $\hat{\Gamma}(x)$ is also a compact set. Now, it follows from Lemma 4.1 that

$$\bigcap_{x\in X}\Gamma(x)\neq\emptyset, $$

which means there exists no $x\in X$ satisfying

$$ \bigl(\alpha(x, \bar{x})f_{1}^{\prime}\bigl(\bar{x}, \eta(x, \bar{x})\bigr)+\rho_{1}(x, \bar{x}), \ldots,\alpha(x, \bar{x})f_{p}^{\prime}\bigl(\bar{x}, \eta(x, \bar{x})\bigr)+ \rho_{p}(x, \bar{x})\bigr)\leqslant 0. $$

Therefore, *x̄* is a solution of (SVVI). □

We end this paper by presenting the following existence theorem for Minty vector variational-like inequality (MVVI). We omit its proof because it can be proven along similar lines of Theorem 4.1.

### Theorem 4.2

*Let* *α*, $\rho_{i}: X\times X\rightarrow \mathbb{R}$, $i\in P$, $\eta: X\times X\rightarrow \mathbb{R}^{n}$. *Assume that*

(i) *for each* $i\in P$, $f_{i}$ *is* $(\alpha,\rho_{i},\eta)$-*monotone on* *X*;
(ii) *α* *and* $\rho_{i}$ *are affine functions with respect to their first argument such that* $\alpha(x,x)=0$ *and* $\rho_{i}(x,x)=0$, $\forall x\in X$;
(iii) *the set*-*valued map* $\Gamma: X\rightarrow2^{X}$ *defined by*
  $$\begin{aligned} \Gamma(x) =&\bigl\{ y\in X: \bigl(\alpha(y, x)f_{1}^{\prime}\bigl(x, \eta(y, x)\bigr)+\rho_{1}(y, x), \ldots, \\ &\alpha(y, x)f_{p}^{\prime} \bigl(x, \eta(y, x)\bigr)+\rho_{p}(y, x)\bigr)\nleqslant 0, \forall x\in X \bigr\} , \end{aligned}$$
  *is closed valued*;
(iv) *there exist a nonempty compact set* $M\subset X$ *and a nonempty compact convex set* $N\subset X$ *such that for each* $y\in X\backslash M$, *there exists* $x\in N$ *such that* $y \notin \Gamma(x)$.

*Then* (MVVI) *is solvable in* *X*.

## Conclusion and remarks

In this paper, we have introduced a kind of generalized invex functions, termed $(\alpha,\rho,\eta)$-invex functions, and Stampacchia and Minty vector variational-like inequalities associated with the introduced extended convexities. Moreover, we have also demonstrated the relationships between the discussed vector variational-like inequalities and nonsmooth multi-objective programming problems involving $(\alpha,\rho,\eta)$-invex functions. Finally, the existence results for Stampacchia and Minty vector variational-like inequalities are proposed by utilizing the KKM-Fan theorem. The results of this note extend some earlier results of Farajzadeh and Lee [9] to a more general class of functions.
